# Supplementary figures and images for: Shifts in water column microbial composition associated to lakes with different trophic conditions: “Lagunas de Montebello” National Park, Chiapas, México
Source: PeerJ. 2022 Sep 16;10:e13999. doi: 10.7717/peerj.13999 (PMC9484458; doi:10.7717/peerj.13999)

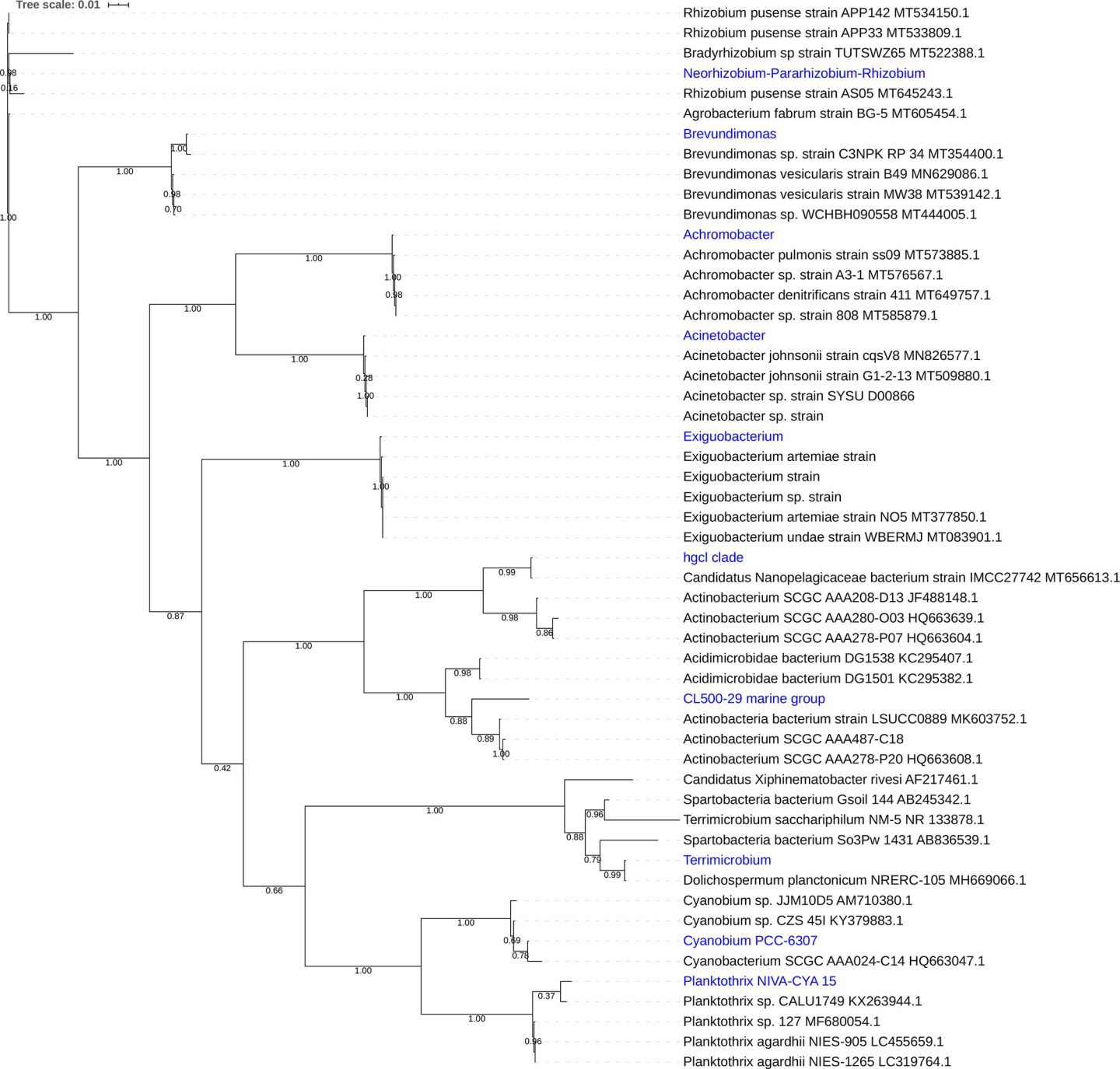

Supplement: Supplemental Information 1 [file peerj-10-13999-s001.pdf]
