# Supplementary material for: Shifts in water column microbial composition associated to lakes with different trophic conditions: “Lagunas de Montebello” National Park, Chiapas, México
Source: PeerJ. 2022 Sep 16;10:e13999. doi: 10.7717/peerj.13999 (PMC9484458; doi:10.7717/peerj.13999)
Supplement: Supplemental Information 2 [file peerj-10-13999-s002.pdf]

S1 Table. Land use difference between 1992 and 2014 for the LMNP*

Land Use 2014	Ha	Percentage (%)
Agriculture	1008.2	15.7
Human settlement	66.4	1
Forest	739	11.5
Body of water	999.1	15.6
Pasture land	349.8	5.5
Vegetation	3248.1	50.7
**PA Total**	**6410.7**	**100**
**Land Use 1992**	**Ha**	**Percentage (%)**
Agriculture	146.6	2.3
Forest	5056.6	78.9
Body of water	653.2	10.2
Pasture land	554.3	8.6
**Total**	**6410.7**	**100**

*To calculate the percentages, the ArcGIS 10.5 program was used, where the land use layer corresponding to the years 1992 and 2014 was entered, later the area in hectares of the polygons was calculated by means of the "Calculate Geometry". The foregoing was done for the area covered by the LMNP. Finally, with the "Summarize" tool, the area of the polygons by attribute of Land Use was calculated, also in hectares.
