# Supplementary material for: Shifts in water column microbial composition associated to lakes with different trophic conditions: “Lagunas de Montebello” National Park, Chiapas, México
Source: PeerJ. 2022 Sep 16;10:e13999. doi: 10.7717/peerj.13999 (PMC9484458; doi:10.7717/peerj.13999)
Supplement: Supplemental Information 3 [file peerj-10-13999-s003.pdf]

S2 Table. Main morphometric parameters and trophic status of the studied Montebello lakes. Depth and thermal type according to Alcocer et al. [19, 25], trophic state according to Vera-Franco et al. [44], and geographic site type according Durán-Calderón et al. [23].

Lake	Lmax km	bmax km	bmean km	A ha	SL km	V km^3	Zmax m	Zmean m	TS	ST
San Lorenzo	3.09	1.29	0.59	181.3	15.01	0.02147	67	11.8	E	P
Bosque Azul	1.32	0.82	0.4	52.5	5.81	0.01050	58	20	E	P
La Encantada	0.39	0.31	0.21	8.2	1.28	0.00241	89	29.4	E	P
Esmeralda	0.14	0.11	0.08	1.1	0.42	0.00004	7	3.6	O	M
Agua Tinta	0.21	0.2	0.14	3	0.65	0.00044	24	14.7	O	M
Ensueño	0.22	0.19	0.12	2.7	0.66	0.00058	35	21.6	O	M
Montebello	1.69	1.14	0.57	96.2	7.84	0.01186	45	12.3	O	M
Tziscao	3.2	1.48	0.96	306.6	13.22	0.08852	86	28.9	O	M
Cinco Lagos	0.82	0.6	0.29	23.7	3.78	0.01006	162	42.5	O	M
Pojoj	1.06	0.74	0.41	43.7	3.61	0.01538	198	35.2	O	M
Dos Lagos	0.34	0.23	0.16	5.2	0.99	0.00132	42	25.2	O	M
Kichail	0.58	0.44	0.21	12.5	2.38	0.00119	22	9.5	O	M

Lmax = maximum length, bmax = maximum width, bmean = mean width, A = surface area, SL = shoreline, V = volume, Zmax = maximum depth, Zmean = mean depth, TS = trophic status, E = eutrophic, O = oligotrophic, ST = site type, P = plateau, M = mountain, (Lakes oriented from NW to SE).
