# Supplementary material for: Shifts in water column microbial composition associated to lakes with different trophic conditions: “Lagunas de Montebello” National Park, Chiapas, México
Source: PeerJ. 2022 Sep 16;10:e13999. doi: 10.7717/peerj.13999 (PMC9484458; doi:10.7717/peerj.13999)
Supplement: Supplemental Information 4 [file peerj-10-13999-s004.pdf]

S3 Table. Water column depths where water samples for Chl-a concentration and microbial community characterization microbiology were obtained

Lake / Z (m)	0	2	3	4	5	9	10	15	18	20	21	25	30	33	34	35	38	40	45	50	55	70	76	84	94	100
San Lorenzo	eu	eu			x		x			a																
Bosque Azul	eu				x		a			a						a										
La Encantada	eu			a			a					a														
Esmeralda	eu		eu/a																							
Agua Tinta	eu				eu		eu	eu		eu																
Ensueño	eu				eu		eu			eu					eu											
Montebello	eu						eu			eu			x					x								
Tziscao central	eu							eu						eu				x	x							
Tziscao puntos	eu						eu								x					a		a				
Tziscao Mirador	eu						eu				a					a										
Cinco Lagos	eu										eu										eu			a		a
Pojoj	eu						eu											eu					a		a	
Dos Lagos	eu					eu			eu				a				a									
Kichail	eu				eu		eu	eu			eu															

eu = sampling depth at euphotic zone, x = sampling depth at aphotic zone, a = sampling depth at anoxia.
