# Supplementary material for: Shifts in water column microbial composition associated to lakes with different trophic conditions: “Lagunas de Montebello” National Park, Chiapas, México
Source: PeerJ. 2022 Sep 16;10:e13999. doi: 10.7717/peerj.13999 (PMC9484458; doi:10.7717/peerj.13999)
Supplement: Supplemental Information 5 [file peerj-10-13999-s005.docx]

**Supplementary** **Table S4.** Permutational Multivariate Analysis of Variance (PERMANOVA) using *adonis*, based on Bray-Curtis distance of the microbial composition of the LMPN. A) Summary of differences within lakes between aerobic and anaerobic zones in the water column; B) along the depth profile; C) between lakes with eutrophic and oligotrophic trophic conditions.

**A)**

| **San Lorenzo** |  |  |  |  |  |  |
| --- | --- | --- | --- | --- | --- | --- |
|  | **Df** | **SSq.** | **Mean SSq.** | **F stat** | ***R*^2^** | ***P*** |
| Group | 1 | 0.029 | 0.029 | 0.188 | 0.059 | 1 |
| Residuals | 3 | 0.475 | 0.158 |  | 0.941 |  |
| Total | 4 | 0.505 |  |  | 1 |  |
|  |  |  |  |  |  |  |
| **Bosque Azul** |  |  |  |  |  |  |
|  | **Df** | **SSq.** | **Mean SSq.** | **F stat** | ***R*^2^** | ***P*** |
| Group | 1 | 0.863 | 0.863 | 6.214 | 0.608 | 0.1 |
| Residuals | 4 | 0.555 | 0.138 |  | 0.391 |  |
| Total | 5 | 1.418 |  |  | 1 |  |
|  |  |  |  |  |  |  |
| **La Encantada** |  |  |  |  |  |  |
|  | **Df** | **SSq.** | **Mean SSq.** | **F stat** | ***R*^2^** | ***P*** |
| Group | 1 | 0.359 | 0.359 | 1.445 | 0.325 |  |
| Residuals | 3 | 0.746 | 0.248 |  | 0.674 | 0.2 |
| Total | 4 | 1.106 |  |  | 1 |  |
|  |  |  |  |  |  |  |
| **Tziscao (Centro, Punto and Mirador)** | | | | | | |
|  | **Df** | **SSq.** | **Mean SSq.** | **F stat** | ***R*^2^** | ***P*** |
| Group | 1 | 0.454 | 0.454 | 1.42 | 0.105 | 0.077 |
| Residuals | 12 | 3.839 | 0.319 |  | 0.849 |  |
| Total | 13 | 4.293 |  |  | 1 |  |
|  |  |  |  |  |  |  |
|  |  |  |  |  |  |  |
| **Cinco lagos** |  |  |  |  |  |  |
|  | **Df** | **SSq.** | **Mean SSq.** | **F stat** | ***R*^2^** | ***P*** |
| Group | 1 | 0.341 | 0.341 | 1.078 | 0.212 | 0.333 |
| Residuals | 4 | 1.264 | 0.316 |  | 0.787 |  |
| Total | 5 | 1.605 |  |  | 1 |  |
|  |  |  |  |  |  |  |
| **Pojoj** |  |  |  |  |  |  |
|  | **Df** | **SSq.** | **Mean SSq.** | **F stat** | ***R*^2^** | ***P*** |
| Group | 1 | 0.413 | 0.413 | 1016 | 0.253 | 0.4 |
| Residuals | 3 | 1.219 | 0.406 |  | 0.746 |  |
| Total | 4 | 1.63 |  |  | 1 |  |
|  |  |  |  |  |  |  |
| **Dos lagos** |  |  |  |  |  |  |
|  | **Df** | **SSq.** | **Mean SSq.** | **F stat** | ***R*^2^** | ***P*** |
| Group | 1 | 0.454 | 0.454 | 2.061 | 0.407 | 0.2 |
| Residuals | 3 | 0.661 | 0.221 |  | 0.592 |  |
| Total | 4 | 1.116 |  |  | 1 |  |

**B)**

| **San Lorenzo** | |  |  |  |  |  |
| --- | --- | --- | --- | --- | --- | --- |
|  | **Df** | **SSq.** | **Mean SSq.** | **F stat** | ***R*^2^** | ***P*** |
| Group | 1 | 0.086 | 0.086 | 0.619 | 0.171 | 0.491 |
| Residuals | 3 | 0.418 | 0.139 |  | 0.828 |  |
| Total | 4 | 0.505 |  |  | 1 |  |
|  |  |  |  |  |  |  |
| **Bosque Azul** | |  |  |  |  |  |
|  | **Df** | **SSq.** | **Mean SSq.** | **F stat** | ***R*^2^** | ***P*** |
| Group | 1 | 0.585 | 0.585 | 2.18 | 0.412 | 0.066 |
| Residuals | 3 | 0.833 | 0.208 |  | 0.587 |  |
| Total | 5 | 1.418 |  |  | 1 |  |
|  |  |  |  |  |  |  |
| **La Encantada** | |  |  |  |  |  |
|  | **Df** | **SSq.** | **Mean SSq.** | **F stat** | ***R*^2^** | ***P*** |
| Group | 1 | 0.411 | 0.411 | 1.777 | 0.371 | 0.158 |
| Residuals | 3 | 0.694 | 0.231 |  | 0.628 |  |
| Total | 4 | 1.106 |  |  | 1 |  |
|  |  |  |  |  |  |  |
| **Esmeralda** |  |  |  |  |  |  |
|  | **Df** | **SSq.** | **Mean SSq.** | **F stat** | ***R*^2^** | ***P*** |
| Group | 1 | 0.252 | 0.252 | 1.015 | 0.252 | 0.4 |
| Residuals | 3 | 0.746 | 0.248 |  | 0.747 |  |
| Total | 4 | 0.998 |  |  | 1 |  |
|  |  |  |  |  |  |  |
| **Agua tinta** |  |  |  |  |  |  |
|  | **Df** | **SSq.** | **Mean SSq.** | **F stat** | ***R*^2^** | ***P*** |
| Group | 1 | 0.176 | 0.176 | 1.101 | 0.268 | 0.416 |
| Residuals | 3 | 0.48 | 0.16 |  | 0.731 |  |
| Total | 4 | 0.657 |  |  | 1 |  |
|  |  |  |  |  |  |  |
| **Ensueño** |  |  |  |  |  |  |
|  | **Df** | **SSq.** | **Mean SSq.** | **F stat** | ***R*^2^** | ***P*** |
| Group | 1 | 0.252 | 0.252 | 1.015 | 0.252 | 0.4 |
| Residuals | 3 | 0.746 | 0.248 |  | 0.747 |  |
| Total | 4 | 0.998 |  |  | 1 |  |
|  |  |  |  |  |  |  |
|  |  |  |  |  |  |  |
| **Montebello** |  |  |  |  |  |  |
|  | **Df** | **SSq.** | **Mean SSq.** | **F stat** | ***R*^2^** | ***P*** |
| Group | 1 | 0.325 | 0.325 | 1.264 | 0.296 | 0.483 |
| Residuals | 3 | 0.773 | 0.257 |  | 0.703 |  |
| Total | 4 | 1.099 |  |  | 1 |  |
|  |  |  |  |  |  |  |
| **Tziscao (Centro, Punto and Mirador)** | | | | | |  |
|  | **Df** | **SSq.** | **Mean SSq.** | **F stat** | ***R*^2^** | ***P*** |
| Group | 1 | 0.366 | 0.366 | 1.121 | 0.085 | 0.291 |
| Residuals | 12 | 3.926 | 0.327 |  | 0.914 |  |
| Total | 13 | 4.293 |  |  | 1 |  |
|  |  |  |  |  |  |  |
| **Cinco Lagos** | |  |  |  |  |  |
|  | **Df** | **SSq.** | **Mean SSq.** | **F stat** | ***R*^2^** | ***P*** |
| Group | 1 | 0.499 | 0.499 | 1.805 | 0.311 | 0.188 |
| Residuals | 4 | 1.106 | 0.276 |  | 0.688 |  |
| Total | 5 | 1.605 |  |  | 1 |  |
|  |  |  |  |  |  |  |
| **Pojoj** |  |  |  |  |  |  |
|  | **Df** | **SSq.** | **Mean SSq.** | **F stat** | ***R*^2^** | ***P*** |
| Group | 1 | 0.52 | 0.52 | 1.402 | 0.318 | 0.166 |
| Residuals | 3 | 1.112 | 0.37 |  | 0.681 |  |
| Total | 4 | 1.63 |  |  | 1 |  |
|  |  |  |  |  |  |  |
| **Dos Lagos** |  |  |  |  |  |  |
|  | **Df** | **SSq.** | **Mean SSq.** | **F stat** | ***R*^2^** | ***P*** |
| Group | 1 | 0.458 | 0.458 | 2.086 | 0.41 | 0.075 |
| Residuals | 3 | 0.658 | 0.219 |  | 0.589 |  |
| Total | 4 | 1.116 |  |  | 1 |  |
|  |  |  |  |  |  |  |
| **Kichail** |  |  |  |  |  |  |
|  | **Df** | **SSq.** | **Mean SSq.** | **F stat** | ***R*^2^** | ***P*** |
| Group | 1 | 0.193 | 0.193 | 0.931 | 0.236 | 0.525 |
| Residuals | 3 | 0.621 | 0.207 |  | 0.763 |  |
| Total | 4 | 0.814 |  |  | 1 |  |

**C)**

| **Trophic state (Eutrophic versus oligotrophic lakes)** | | | | | | |
| --- | --- | --- | --- | --- | --- | --- |
|  | **Df** | **SSq.** | **Mean SSq.** | **F stat** | ***R*^2^** | ***P*** |
| Group | 1 | 4.479 | 4.479 | 14.017 | 0.175 | 0.0009* |
| Residuals | 66 | 21.09 | 0.3195 |  | 0.824 |  |
| Total | 67 | 25.569 |  |  | 1 |  |
