# Supplementary material for: Shifts in water column microbial composition associated to lakes with different trophic conditions: “Lagunas de Montebello” National Park, Chiapas, México
Source: PeerJ. 2022 Sep 16;10:e13999. doi: 10.7717/peerj.13999 (PMC9484458; doi:10.7717/peerj.13999)
Supplement: Supplemental Information 6 — A) Summary of differences within lakes between aerobic and anaerobic zones in the water column; B) along the depth profile; C) between lakes with eutrophic and oligotrophic conditions. [file peerj-10-13999-s006.pdf]

Supplementary Table S5. Permutational Multivariate Analysis of Variance (PERMANOVA) using *adonis*, based on Bray-Curtis distance of the microbial composition of the LMPN. A) Summary of differences within lakes between aerobic and anaerobic zones in the water column ; B) along the depth profile ; C) between lakes with eutrophic and oligotrophic conditions.

A)

	**Df**	**SSq.**	**Mean SSq.**	**F stat**	R^2	***P***
Group	1	0.029	0.029	0.188	0.059	1
Residuals	3	0.475	0.158		0.941	
Total	4	0.505			1	

Bosque Azul

	**Df**	**SSq.**	**Mean SSq.**	**F stat**	R^2	***P***
Group	1	0.863	0.863	6.214	0.608	0.1
Residuals	4	0.555	0.138		0.391	
Total	5	1.418			1	

La Encantada

	**Df**	**SSq.**	**Mean SSq.**	**F stat**	R^2	***P***
Group	1	0.359	0.359	1.445	0.325	
Residuals	3	0.746	0.248		0.674	0.2
Total	4	1.106			1	

Tziscao (Centro, Punto and Mirador)

	**Df**	**SSq.**	**Mean SSq.**	**F stat**	R^2	***P***
Group	1	0.454	0.454	1.42	0.105	0.077
Residuals	12	3.839	0.319		0.849	
Total	13	4.293			1	

Cinco lagos

	Df	SSq.	Mean SSq.	F stat	R^2	*P*
Group	1	0.341	0.341	1.078	0.212	0.333
Residuals	4	1.264	0.316		0.787	
Total	5	1.605			1	

Pojoj

	Df	SSq.	Mean SSq.	F stat	R^2	*P*
Group	1	0.413	0.413	1016	0.253	0.4
Residuals	3	1.219	0.406		0.746	
Total	4	1.63			1	

Dos lagos

	Df	SSq.	Mean SSq.	F stat	R^2	*P*
Group	1	0.454	0.454	2.061	0.407	0.2
Residuals	3	0.661	0.221		0.592	
Total	4	1.116			1	

B)

San Lorenzo

	Df	SSq.	Mean SSq.	F stat	R^2	*P*
Group	1	0.086	0.086	0.619	0.171	0.491
Residuals	3	0.418	0.139		0.828	
Total	4	0.505			1	

Bosque Azul

	Df	SSq.	Mean SSq.	F stat	R^2	*P*
Group	1	0.585	0.585	2.18	0.412	0.066
Residuals	3	0.833	0.208		0.587	
Total	5	1.418			1	

La Encantada

	Df	SSq.	Mean SSq.	F stat	R^2	*P*
Group	1	0.411	0.411	1.777	0.371	0.158
Residuals	3	0.694	0.231		0.628	
Total	4	1.106			1	

Esmeralda

	Df	SSq.	Mean SSq.	F stat	R^2	*P*
Group	1	0.252	0.252	1.015	0.252	0.4
Residuals	3	0.746	0.248		0.747	
Total	4	0.998			1	

Agua tinta

	Df	SSq.	Mean SSq.	F stat	R^2	*P*
Group	1	0.176	0.176	1.101	0.268	0.416
Residuals	3	0.48	0.16		0.731	
Total	4	0.657			1	

Ensueño

	Df	SSq.	Mean SSq.	F stat	R^2	*P*
Group	1	0.252	0.252	1.015	0.252	0.4
Residuals	3	0.746	0.248		0.747	
Total	4	0.998			1	

Montebello

	Df	SSq.	Mean SSq.	F stat	R^2	*P*
Group	1	0.325	0.325	1.264	0.296	0.483
Residuals	3	0.773	0.257		0.703	
Total	4	1.099			1	

Tziscao (Centro, Punto and Mirador)

	Df	SSq.	Mean SSq.	F stat	R^2	*P*
Group	1	0.366	0.366	1.121	0.085	0.291
Residuals	12	3.926	0.327		0.914	
Total	13	4.293			1	

Cinco Lagos

	Df	SSq.	Mean SSq.	F stat	R^2	*P*
Group	1	0.499	0.499	1.805	0.311	0.188

Residuals	4	1.106	0.276		0.688	
Total	5	1.605			1	

Pojoj

	Df	SSq.	Mean SSq.	F stat	R^2	*P*
Group	1	0.52	0.52	1.402	0.318	0.166
Residuals	3	1.112	0.37		0.681	
Total	4	1.63			1	

Dos Lagos

	Df	SSq.	Mean SSq.	F stat	R^2	*P*
Group	1	0.458	0.458	2.086	0.41	0.075
Residuals	3	0.658	0.219		0.589	
Total	4	1.116			1	

Kichail

	Df	SSq.	Mean SSq.	F stat	R^2	*P*
Group	1	0.193	0.193	0.931	0.236	0.525
Residuals	3	0.621	0.207		0.763	
Total	4	0.814			1	

C)

Trophic state (Eutrophic versus oligotrophic lakes)						
	**Df**	**SSq.**	**Mean SSq.**	**F stat**	***R^2***	***P***
Group	1	4.479	4.479	14.017	0.175	0.0009*
Residuals	66	21.09	0.3195		0.824	
Total	67	25.569			1	
